# Upregulation of cancer-associated myofibroblasts by TGF-*β* from scirrhous gastric carcinoma cells

**DOI:** 10.1038/bjc.2011.330

**Published:** 2011-08-23

**Authors:** Y Fuyuhiro, M Yashiro, S Noda, S Kashiwagi, J Matsuoka, Y Doi, Y Kato, T Hasegawa, T Sawada, K Hirakawa

**Affiliations:** 1 Department of Surgical Oncology, Osaka City University Graduate School of Medicine, 1-4-3 Asahi-machi, Abeno-ku, Osaka 545-8585, Japan; 2 Oncology Institute of Geriatrics and Medical Science, Osaka City University Graduate School of Medicine, Osaka, Japan

**Keywords:** myofibroblasts, cancer-associated fibroblasts, TGF-*β*, scirrhous gastric carcinoma, microenvironment, interaction

## Abstract

**Background:**

Myofibroblasts in the cancer microenvironment have recently been implicated in tumour growth and metastasis of gastric cancer. However, the mechanisms responsible for the regulation of myofibroblasts in cancer-associated fibroblasts (CAFs) remain unclear. This study was performed to clarify the mechanisms for regulation of myofibroblasts in gastric cancer microenvironment.

**Methods:**

Two CAFs (CaF-29 and CaF-33) from the tumoural gastric wall and a normal fibroblast (NF-29) from the nontumoural gastric wall, 4 human gastric cancer cell lines from scirrhous gastric cancer (OCUM-2MD3 and OCUM-12), and non-scirrhous gastric cancer (MKN-45 and MKN-74) were used. Immunofluorescence microscopy by triple-immunofluorescence labelling (*α*-SMA, vimentin, and DAPI) was performed to determine the presence of *α*-SMA-positive myofibroblasts. Real-time RT–PCR was performed to examine *α-SMA* mRNA expression.

**Results:**

Immunofluorescence microscopy showed that the frequency of myofibroblasts in CaF-29 was greater than that in NF-29. The number of myofibroblasts in gastric fibroblasts gradually decreased with serial passages. Transforming growth factor-*β* (TGF-*β*) significantly increased the *α-SMA* expression level of CAFs. Conditioned medium from OCUM-2MD3 or OCUM-12 cells upregulated the *α*-SMA expression level of CAFs, but that from MKN-45 or MKN-74 cells did not. The *α-SMA* upregulation effect of conditioned medium from OCUM-2MD3 or OCUM-12 cells was significantly decreased by an anti-TGF-*β* antibody or *Smad2* siRNA.

**Conclusion:**

Transforming growth factor-*β* from scirrhous gastric carcinoma cells upregulates the number of myofibroblasts in CAFs.

Recently, tumour progression has been recognised as the product of evolving crosstalk between cancer cells and the surrounding tissue (Kalluri and Zeisberg, 2006). The normal stroma contains few fibroblasts, but there is a dramatic increase in fibroblast-like cells within the reactive stroma surrounding inflamed or neoplastic tissue (Worthley *et al*, 2010). Cancer cells themselves may alter their adjacent stroma to form a permissive and supportive environment for tumour progression (Durning *et al*, 1984; Schor *et al*, 1988). Fibroblasts within the tumour stroma, known as carcinoma-associated fibroblasts (CAFs), including both fibroblasts and myofibroblasts (Semba *et al*, 2009), play a critical role in the regulation of tumour growth (Kalluri and Zeisberg, 2006; Guo *et al*, 2008; Noma *et al*, 2008; Shimoda *et al*, 2010; Yashiro and Hirakawa, 2010). Myofibroblasts, which are distinct from fibroblasts in their expression of both vimentin and *α*-smooth muscle actin (*α*-SMA), have recently been implicated in important aspects of solid tumour progression (Olumi *et al*, 1999; Hasebe *et al*, 2000; Tomasek *et al*, 2002; Kalluri and Zeisberg, 2006; Tsujino *et al*, 2007; Matsubara *et al*, 2009), because myofibroblasts produce a number of important factors that can directly promote growth in the adjacent epithelium (Kalluri and Zeisberg, 2006; Brenmoehl *et al*, 2009). Scirrhous gastric cancer cells proliferate with fibrosis when the cancer cells invade into the submucosa containing abundant stromal cells (Nakazawa *et al*, 2003). We have previously reported that gastric fibroblasts play an important role in the progression, growth, and spread of scirrhous gastric cancers (Yashiro and Hirakawa, 2010), and myofibroblasts in gastric fibroblasts are particularly associated with scirrhous-type gastric cancer and the poor prognosis of gastric cancer patients (Kinugasa *et al*, 1998; Otsuji *et al*, 2004).

Overexpression of transforming growth factor-*β* (TGF-*β*) is reported to be correlated with a poor prognosis for gastric tumours (Naef *et al*, 1997; Maehara *et al*, 1999; Saito *et al*, 2000), especially scirrhous gastric carcinoma (Kinugasa *et al*, 1998; Hawinkels *et al*, 2007), suggesting that TGF-*β* signalling might have an important role in the progression of scirrhous gastric cancer cells (Inoue *et al*, 1997; Kinugasa *et al*, 1998; Kawajiri *et al*, 2008). Transforming growth factor-*β* activates type II TGF-*β* receptors (T*β*R-II), which phosphorylate type I TGF-*β* receptors (T*β*R-I) (Heldin *et al*, 1997; Massague, 2008). Activated T*β*R-I kinase phosphorylates Smad2/3. Phosphorylated Smad2/3 is associated with Smad4 and translocation in the nucleus as transcriptional factors. Transforming growth factor-*β* remains among the key factors responsible for the development of a myofibroblastic phenotype from a variety of precursor cells, including fibroblasts (Tomasek *et al*, 2002; Webber *et al*, 2010). However, the mechanisms responsible for the upregulation of myofibroblasts remain unclear.

In this study, we investigated the effect of gastric cancer cells on normal fibroblasts and CAFs isolated from the primary tumour site to understand the mechanisms for regulation of myofibroblast expression in the cancer microenvironment.

## Materials and methods

### Cell culture and cell lines

We used three human gastric fibroblast cell lines and four human gastric cancer cell lines in this study. Fibroblasts cell lines were established at our department. The NF-29 and CAF-29 were established from a 68-year-old male patient with poorly differentiated gastric carcinoma who had a total gastrectomy. The NF-29 was from nontumoural gastric wall, and CaF-29 was from tumoural gastric wall. The CaF-33 was established from a 65-year-old male patient with poorly differentiated gastric carcinoma who had a distal gastrectomy. The primary culture was initiated as follows: the primary tumour was excised under aseptic conditions, and minced with forceps and scissors. The tumour pieces were cultivated in Dulbecco's modified Eagle medium (DMEM; Nikken, Kyoto, Japan) with 10% heat-inactivated fetal calf serum (FCS; Life Technologies, Inc., Grand Island, NY, USA), 100 IU ml^–1^ penicillin (ICN Biomedical, Costa Mesa, CA, USA), 100 *μ*g ml^–1^ streptomycin (ICN Biomedical), and 0.5 mM sodium pyruvate (Cambrex, Walkersville, MD, USA), and incubated in humidified incubators at 37 °C in an atmosphere of 5% CO_2_ in air. The fibroblasts initially grew in a monolayer. After ∼2 weeks, fibroblasts were collected and transferred to another culture dish. Serial passages were then carried out every 4–7 days. The fibroblasts were used 3–12th passage in culture. Four human gastric cancer cell lines, including OCUM-2MD3 (poorly differentiated adenocarcinoma) (Yashiro *et al*, 1996), OCUM-12 (poorly differentiated adenocarcinoma) (Kato *et al*, 2010), MKN-45 (poorly differentiated adenocarcinoma) (Motoyama *et al*, 1986), and MKN-74 (well-differentiated adenocarcinoma) (Motoyama *et al*, 1986) were seeded in a 100-mm dish (Falcon, Lincoln Park, NJ, USA) and cultured. OCUM-2MD3 and OCUM-12 were derived from scirrhous gastric carcinoma.

### Immunofluorescence microscopy

To examine incubating myofibroblast content of fibroblast, immunofluorescence microscopy was performed. Triple-immunofluorescence labelling was performed to examine the presence of *α*-SMA-positive myofibroblasts. Fibroblasts were washed twice with Dulbecco’s PBS and fixed with acetone for 5 min, and then blocked with 3% BSA (diluted in PBS) for 30 min at room temperature. Fibroblasts were further incubated with anti-human *α*-SMA antibody (R&D Systems, Minneapolis, MN, USA; 1 : 100) and vimentin (Santa Cruz, Santa Cruz, CA, USA; 1 : 50) and DAPI (Wako, Osaka, Japan; 1 : 10 000) for 60 min at room temperature. Fibroblasts were viewed under a fluorescence microscope Leica Digital Microscopy DMI 6000 (Leica Microsystems, Heidelberg, Germany) with a DAPI filter (365 nm excitation), *α*-SMA fluorescence with a PE filter (546 nm excitation), and vimentin with a FITC filter (450–490 nm excitation). Cells that were *α*-SMA positive were determined as myofibroblasts. The percentage of binding cells was calculated as follows: (number of myofibroblasts/number of total cells) × 100. The percentage of *α*-SMA-positive myofibroblast cells was determined in 10 random fields. At least, three independent experiments were performed.

### Western blot analysis

Fibroblasts were rinsed with PBS and were lysed in a lysis buffer. Aliquots containing 30 *μ*g of total protein were subjected to SDS–PAGE, and the protein bands were transferred to a polyvinylidene difluoride membrane (Amersham, Aylesbury, UK). The membrane was placed in the TBS-T solution containing the primary antibody, *α*-SMA (Dako, Glostrup, Denmark; 1 : 1000) or *β*-Actin (Cell Signaling, Danvers, MA, USA; 1 : 1000), and allowed to react at 4 °C overnight for western blotting. The bands were detected using an enhanced chemiluminescence system (Amersham). An immunoblot analysis was performed twice.

### Preparation of conditioned medium

Conditioned medium from gastric cancer cells was prepared as follows. Gastric cancer cells (5 × 10^4^ cells ml^–1^) were seeded into 100-mm plastic dishes with 10 ml of DMEM containing 2% FCS and incubated for 3 days. The number of fibroblasts and gastric cancer cells in each dish was ∼2.5 × 10^6^ cells after 3 days of incubation. To obtain conditioned medium, fibroblasts and gastric cancer cells were washed twice with PBS and then incubated for 3 days in 3 ml of DMEM. Conditioned medium was collected from each dish and centrifuged at 1000 **g** for 5 min. The supernatant was stored as conditioned medium at −20 °C until use. As a control, DMEM was used instead of conditioned medium.

### Quantitative real-time reverse transcriptase-polymerase chain reaction (RT–PCR)

Real-time RT–PCR was performed to examine *α-SMA* mRNA expression. Gastric cancer cells and fibroblasts were incubated in 3 ml DMEM containing 2% FCS with 50% each conditioned medium. After 3 days of incubation, the total cellular RNA was extracted using Trizol reagent (Invitrogen, Carlsbad, CA, USA). After removal of genomic DNA by DNAse, cDNA was prepared from 20 *μ*g RNA using random primers (Invitrogen). To determine fold changes in each gene, real-time RT–PCR was performed on the ABI Prism 7000 (Applied Biosystems, Foster City, CA, USA), using commercially available gene expression assays for *α-SMA* (Hs00426835). *Glyceraldehyde-3-phosphate dehydrogenase* (*GAPDH*) was used as an internal standard to normalise mRNA levels. The threshold cycle (*C*_t_) values were used to calculate the relative expression ratios between control and treated cells using the formula described by Pfaffl (Pfaffl, 2001). The *α-SMA* expression level was calculated relative to that of NF-29 at third passage (1.0-fold as the control). Quantitative RT–PCR reactions were performed in triplicate.

### Effect of conditioned medium, TGF-*β*, or anti-TGF-*β* neutralising antibody on *α-SMA* expression of fibroblasts

Fibroblasts were incubated in 3 ml DMEM containing 2% FCS with 50% of each conditioned medium, 10 ng ml^–1^ TGF-*β* (R&D Systems), and 10 *μ*M anti-TGF-*β* neutralising antibody. After 3 days of incubation, *α-SMA* expression of fibroblasts was examined by RT–PCR as described above.

### The effect of Smad2 siRNA on *α-SMA* expression of fibroblasts

The sequences for *Smad2* small interfering RNA (siRNA) are designed as: *Smad2* siRNA sense, 5′-GUCCCAUGAAAAGACUUAATT-3′ antisense, 5′-UUAAGUCUUUUCAUGGGACTT-3′. Control non-targeting siRNA was purchased from Ambion (Austin, TX, USA). The transfection mixture was prepared by incubating 5 *μ*l of siPORT Neo-Fx (Ambion) and 295 *μ*l of Opti-MEMI. The CAF-33 cells were prepared at 50–60% confluence in six-well dishes. The transfection mixture (final siRNA concentration was 30 nM) was added to six-well dish containing 2 ml of DMEM with 10% FBS. At 24 h after transfection, CAF-33 cells were incubated in addition of conditioned medium from gastric cancer cells. After 3 days of incubation, the total cellular RNA was extracted, and RT–PCR was performed.

### Statistical analysis

Data are expressed as the means±s.d. from at least three independent determinations. Significance of difference was analysed using unpaired Student’s *t-*tests. Values of *P*<0.05 were considered to indicate statistical significance.

## Results

### The proportion of myofibroblasts in the primary culture

**Figure 1:**
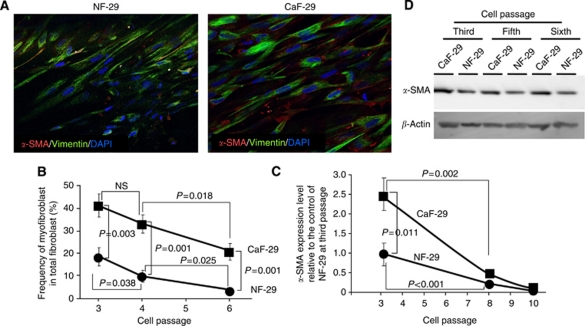
The *α*-smooth muscle actin (*α*-SMA) expression in fibroblasts. (**A**) Immunofluorescence of NF-29 fibroblasts and CaF-29 fibroblasts. Fibroblasts were stained with *α*-SMA (red), vimentin (green), and cell nuclei were stained with DAPI (blue). The percentage of myofibroblasts accompanying cancer-associated fibroblasts, CaF-29, from gastric tumour lesions was higher than that from normal fibroblasts, NF-29, derived from normal gastric tissue. (**B**) The proportion of myofibroblasts in the primary culture. The percentage of *α*-SMA-positive myofibroblast cells was determined in 10 random fields. The percentage of *α*-SMA-positive myofibroblasts cells of NF-29 (•) and CaF-29 (▪) at the third passage were 42% and 18%, respectively. At the fourth or sixth passage, the myofibroblast contents of both NF-29 and CaF-29 fibroblast cultures were lower than that at the third passage. (**C**) The expression level of *α-SMA* mRNA in the primary culture. The *α-SMA* expression level of CaF-29 (•) at the third passage was 2.5 relative to the *α-SMA* expression level of NF-29 (▪) as the control. The *α-SMA* expression level of both NF-29 and CaF-29 at the eighth or tenth passage was under 0.3 relative to the control of NF-29 at the third passage. (**D**) Western blot analysis. The *α*-SMA expression level in CaF-29 was higher than that of NF-29 at each passage. The *α*-SMA expression level in CaF-29 at the third passage was high in comparison with that at fifth or sixth passage.

Immunofluorescence microscopy showed that NF-29 and CaF-29 fibroblasts contained *α*-SMA-positive (red) myofibroblast cells. A larger number of *α*-SMA-positive myofibroblasts were found in CaF-29 cells than in NF-29 cells. All cultured fibroblasts at the third passage were vimentin positive (green; Figure 1A). The ratios of myofibroblasts among the total fibroblasts in CaF-29 and NF-29 cultures at the third passage were 42% and 18%, respectively. The ratio of myofibroblasts in CaF-29 was significantly greater than that in NF-29 at the third (*P*=0.003), fourth (*P*=0.001), and sixth (*P*=0.001) passages. With each serial passage, the frequency of myofibroblasts in CaF-29 or NF-29 decreased, such that the frequency of myofibroblasts at the sixth passage was significantly decreased in CaF-29 (*P*=0.018) or NF-29 (*P*=0.025) compared with that at the fourth passage (Figure 1B). The *α-SMA* mRNA expression level of the cancer-associated fibroblast, CaF-29, was significantly (*P*=0.011) higher than that of the normal NF-29 fibroblasts at third passage. With each serial passage, the *α-SMA* expression level in CaF-29 or NF-29 decreased, and that in CaF-29 (*P*=0.002) or NF-29 (*P*<0.001) at the eighth passage was significantly lower in comparison with that at the third passage. There was no significant difference between the *α-SMA* expression levels of CaF-29 and NF-29 at the eighth and tenth passages (Figure 1C). Western blot analysis also showed that *α*-SMA expression level in CaF-29 was higher than that of NF-29 at each passage. The *α*-SMA expression level in CaF-29 at the third passage was high in comparison with that at other passages (Figure 1D).

### Effect of conditioned medium from gastric cancer cells on *α-SMA* expression of fibroblasts

**Figure 2:**
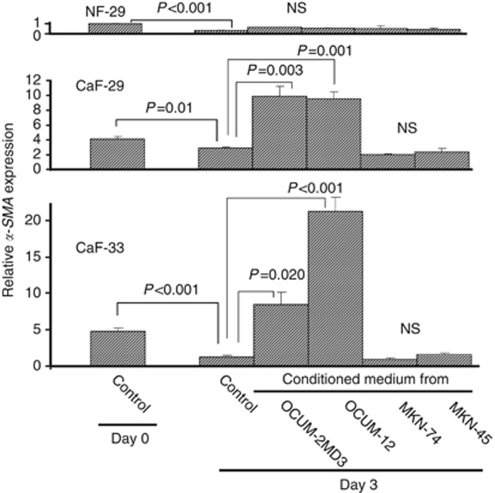
Effect of conditioned medium from gastric cancer cells on the *α-SMA* expression of fibroblast. The *α-SMA* expression levels of cancer-associated fibroblasts CaF-29 and CaF-33 were significantly increased by conditioned medium from scirrhous gastric cancer cells OCUM-2MD3 and OCUM-12, but not by conditioned medium from non-scirrhous gastric cancer cells MKN-45 and MKN-74. The *α-SMA* expression of normal NF-29 fibroblasts was not increased by the addition of conditioned medium from any gastric cancer cells. The graph depicts expression levels relative to control NF-29 fibroblasts at day 0.

The *α-SMA* expression level of the controls at day 3 was significantly decreased compared with that at day 0 in NF-29 (*P*<0.001), CaF-29 (*P*=0.01), and CaF-33 (*P*<0.001) cells. Conditioned medium from OCUM-2MD3 and OCUM-12 cells significantly increased the *α-SMA* expression level of CaF-29 and CaF-33 cells, but not that from MKN-45 and MKN-74 cells. The *α-SMA* expression level of NF-29 cells was not increased by any conditioned medium from gastric cancer cells. The *α-SMA* expression level of NF-29 at day 0 was set as 1 (Figure 2).

### Effect of TGF-*β* or Smad2 siRNA on *α-SMA* expression of fibroblasts

**Figure 3:**
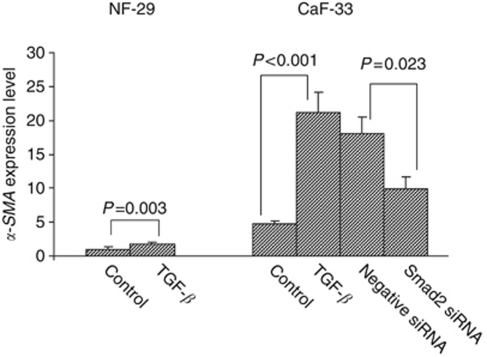
Effect of TGF-*β* or Smad2 siRNA on *α-SMA* expression of fibroblasts. The TGF-*β* (10 ng ml^–1^) increased the *α-SMA* expression level of gastric fibroblasts. The upregulation of *α-SMA* expression level by TGF-*β* was significantly (*P*=0.023) decreased by *Smad2* siRNA (30 nM) in CaF-33 cells. The graph depicts expression levels relative to control NF-29.

Transforming growth factor-*β* significantly upregulated the *α-SMA* expressions of both NF-29 and CaF-33, whereas the *α-SMA* expression level of CaF-33 by TGF-*β* was higher than that of NF-29 by TGF-*β*. The TGF-*β*-stimulating effect of *α-SMA* expression in CaF-33 cells was significantly (*P*=0.023) decreased by *Smad2* siRNA (30 nM) compared with those treated by negative control siRNA (Figure 3).

### Effect of anti-TGF-*β* antibody or Smad2 siRNA on *α-SMA-*stimulating effect of conditioned medium

**Figure 4:**
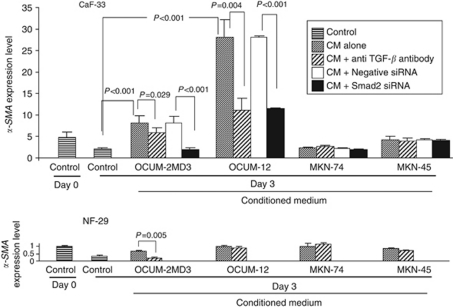
Effect of anti-TGF-*β* antibody or Smad2 siRNA on *α-SMA* expression of fibroblasts. The conditioned medium (CM) from OCUM-2MD3 and OCUM-12 cells significantly increased the *α-SMA* expression level of cancer-associated fibroblast CaF-33 cells. The *α-SMA* expression level of CaF-33 with the addition of CM of OCUM-2MD3 and OCUM-12 was significantly decreased by anti-TGF-*β* antibody or Smad2 siRNA (30 nM). In contrast, CM from MKN-45 and MKN-74 did not affect the *α-SMA* expression level of CaF-33. The graph depicts expression levels relative to control NF-29 fibroblasts at day 0.

Anti-TGF-*β* antibody significantly decreased the *α-SMA* expression level of CaF-33 that was upregulated by conditioned medium from OCUM-2MD3 or OCUM-12 cells. The *Smad2* siRNA (30 nM) significantly (*P*<0.001) decreased the *α-SMA* expression level of CAF-33 that was upregulated by conditioned medium from OCUM-2MD3 or OCUM-12 cells. In contrast, no difference of *α-SMA* expression level between *Smad2* siRNA and negative siRNA treatment was found in CAF-33 cells with conditioned medium from MKN-45 or MKN-74. In NF-29 cells with conditioned medium from OCUM-2MD3 cells, the anti-TGF-*β* antibody significantly decreased the *α-SMA* expression level (Figure 4).

## Discussion

The *α*-SMA expression is reported to be the most common marker for myofibroblast identification and allows the monitoring of the behaviour of this cell (Desmouliere *et al*, 2004), whereas there is no myofibroblast-specific immunocytochemical marker (De Wever *et al*, 2008). In this study, we defined myofibroblasts based on a combination of positive markers, both *α*-SMA and vimentin. All cultured fibroblasts at third passage were vimentin positive, which suggested that no epithelial cells were contained in the culture cells. The rate of myofibroblasts in CAFs derived from gastric tumours was greater than that in fibroblasts derived from normal gastric tissue. The number of myofibroblasts gradually decreased with serial passage in the normal tissue culture. Conditioned medium from OCUM-2MD3 or OCUM-12 cells upregulated the *α*-SMA expression level of CAFs. These findings might suggest that myofibroblasts are reversible to fibroblasts and that some factor(s) from scirrhous gastric cancer cells maintain the myofibroblast phenotype in CAFs. In contrast, conditioned medium from gastric cancer cells did not affect the *α*-SMA expression level of normal NF-29 fibroblasts, suggesting different responses of the *α*-SMA phenotype to conditioned medium for CAFs and normal fibroblasts. Future studies might be needed to determine which molecules of CAFs represent the myofibroblast phenotype in comparison with normal host fibroblasts.

Conditioned medium from scirrhous gastric cancer cells (OCUM-2MD3 and OCUM-12) significantly increased the number of myofibroblasts in CAFs, whereas conditioned medium from non-scirrhous gastric cancer cells (MKN-45 and MKN-74) did not; gastric cancer cells of varying differentiation had differential effects on the phenotypic features of fibroblasts. Scirrhous gastric cancer cells proliferate diffusely with extensive fibrosis, whereas most intestinal-type carcinoma cells proliferate with fewer stromal cells (Japanese Gastric Cancer, 1998). This histological difference in the volume of the stroma might be determined by the response of gastric fibroblasts to factor(s) from gastric cancer cells. Myofibroblasts represent an important prognostic factor for invasive growth that translates into a poor clinical prognosis for patients with various types of cancer (Eyden *et al*, 2009; Worthley *et al*, 2010; Yamashita *et al*, 2010). Myofibroblasts induced by scirrhous gastric cancer cells may create a congenial environment for the progression of scirrhous gastric carcinoma.

Transforming growth factor-*β* is secreted by a range of tumour cells (Mueller and Fusenig, 2004) and mediates the interaction of cancer cells with stromal fibroblasts (Kalluri and Zeisberg, 2006). Webber *et al* (2010) found that some cancer-derived exosomes could trigger elevated *α*-SMA expression and other changes consistent with the process of fibroblast differentiation into myofibroblasts. It has been reported that the cancer cell-derived TGF-*β* modulates myofibroblast differentiation in colon cancer (De Wever *et al*, 2004), breast cancer (Casey *et al*, 2008), and squamous cancer (Lewis *et al*, 2004). In this study, we found that the number of myofibroblasts of gastric fibroblasts was also upregulated by TGF-*β*. Moreover, our study indicated that the number of myofibroblasts was more increased by TGF-*β* in cancer-associated fibroblasts in comparison with normal fibroblasts. In this study, TGF-*β* significantly increased the *α-SMA* expression level of CAFs, and the *α-SMA* upregulation effects of conditioned medium was significantly decreased by an anti-TGF-*β* antibody and *Smad2* siRNA. These findings suggested that TGF-*β* produced by tumour cells may contribute to maintaining the myofibroblastic phenotype and might play an important role in the malignant phenotype in the cancer microenvironment. Previous studies have reported that in clinical cases gastric tumours with overexpression of TGF-*β* imply a poor prognosis, and that TGF-*β* expression levels are higher in scirrhous gastric cancer cells than in non-scirrhous gastric cancer cells (Mahara *et al*, 1994; Kinugasa *et al*, 1998; Hawinkels *et al*, 2007). These findings might explain one of the mechanisms for the different responses of CAFs to conditioned medium from gastric cancer cells.

Transforming growth factor-*β* is synthesised as an inactive precursor, the large latent complex consisting of a TGF-*β* dimer, the latency-associated protein (LAP) and latent TGF-*β-*binding protein (LTBP) for localisation and binding to the ECM (Hawinkels *et al*, 2007). Before TGF-*β* can exert its biological effects, LAP and LTBP have to be dissociated. The urokinase plasminogen activator (uPA) is one factor that can activate latent TGF-*β*. We previously reported that scirrhous gastric cancer cells produced higher amounts of uPA (Yashiro *et al*, 1995). Hawinkels *et al* (2007) also observed a significant relation between active TGF-*β* levels and urokinase activity, implying plasmin, via urokinase-mediated plasminogen activation, as a principal candidate of latent TGF-*β* activation. The correlation between scirrhous gastric cancer and uPA suggests a role for plasmin in TGF-*β* activation in the tumour-specific microenvironment, resulting in transformation of resident fibroblasts to tumour-promoting myofibroblasts (Hawinkels *et al*, 2007). These findings might be one of the reasons for the high frequency of myofibroblasts from cancer tissue in comparison with that from normal tissue in scirrhous gastric cancer.

Myofibroblasts are a contractile cell types with actin expression (Hinz *et al*, 2001). Scirrhous gastric carcinomas sometimes cause a rapid contraction of the stomach wall at an advanced stage, the so-called ‘linitis plastica’. Ura *et al* (1991) reported that the activated form of TGF-*β* might contract the stomach wall. The upregulation of myofibroblasts by TGF-*β* released from scirrhous gastric carcinoma cells might explain the mechanisms underlying contraction of the stomach wall in cases of linitis plastica.

De Wever *et al* (2008) reported the multiplicity of molecules involved in the interaction between cancer cells and (myo)fibroblasts. Although TGF-*β* is a dominant indirect proinvasive factor for epithelial cancer cells, other factors acting in combination may also be implicated (De Wever *et al*, 2008). Future studies might be needed to determine which molecules of CAFs represent the myofibroblast phenotype in comparison with normal host fibroblasts.

Myofibroblasts produce a number of important factors that can directly promote growth in the adjacent epithelium (Kalluri and Zeisberg, 2006; Brenmoehl *et al*, 2009). The relationship between cancer cells and myofibroblasts in the tumour microenvironment might be an important target for new therapeutic approaches in controlling the growth and metastasis of cancer. The increase in myofibroblasts within the cancer microenvironment may occur through TGF-*β* signalling. The TGF-*β* receptor might therefore be a promising target molecule for cancer therapy in the gastric cancer–stroma interaction, especially in the scirrhous type of cancer.

In conclusion, TGF-*β* from scirrhous gastric carcinoma cells upregulates the proportion of cancer-associated myofibroblasts.
